# Supplementary material for: Visual Word Form Area demonstrates individual and task-agnostic consistency but inter-individual variability
Source: bioRxiv. 2025 Jul 27:2025.07.23.666206. Preprint. [Version 1] doi: 10.1101/2025.07.23.666206 (PMC12330508; doi:10.1101/2025.07.23.666206)
Supplement: Supplement 1 [file media-1.pdf]

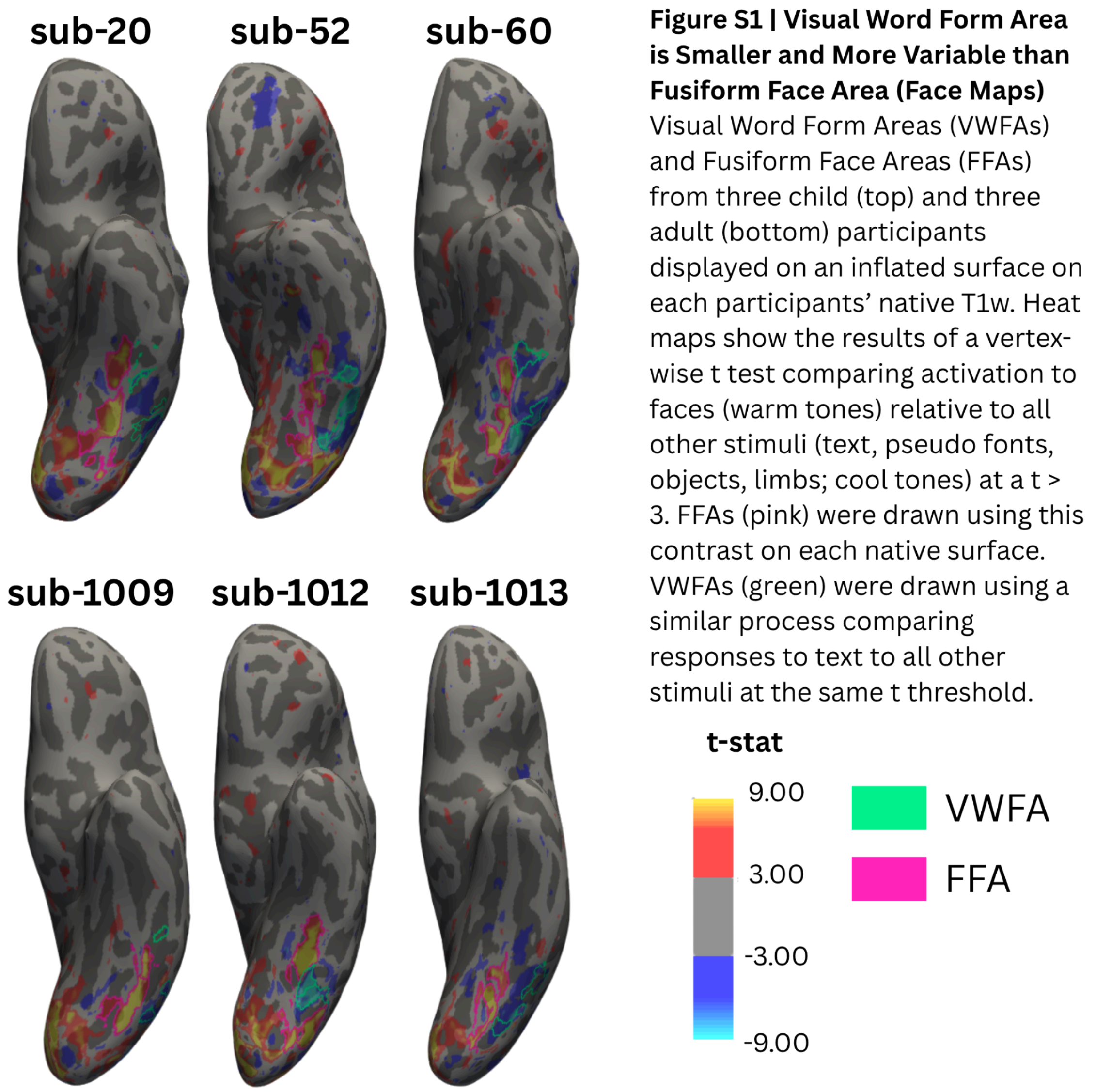

Figure S1 | Visual Word Form Area is Smaller and More Variable than Fusiform Face Area (Face Maps)

Visual Word Form Areas (VWFAs) and Fusiform Face Areas (FFAs) from three child (top) and three adult (bottom) participants displayed on an inflated surface on each participants' native T1w. Heat maps show the results of a vertex-wise t test comparing activation to faces (warm tones) relative to all other stimuli (text, pseudo fonts, objects, limbs; cool tones) at a $t > 3$. FFAs (pink) were drawn using this contrast on each native surface. VWFAs (green) were drawn using a similar process comparing responses to text to all other stimuli at the same t threshold.

SepTask VWFA Dice Similarity Coefficient			
	**Mean DSC**	**SD**	**SEM**
All Participants	0.465	0.262	0.031
Children Only	0.430	0.260	0.034
Adults Only	0.618	0.207	0.057

Table S1 | Dice Similarity Calculations for Split Task Visual Word Form Area
Results from a Dice Similarity calculation for computing the overlap of the VWFA when drawn using only one-back task data and only fixation task data.

SepTask VWFA Size Difference						
	**t**	**CI**		**DOF**	***p***	
	(ob > fix)	*low*	*high*			
All Participants	**2.416**	**0.029**	**0.298**	**95**	**0.018**	*
Children Only	**2.358**	**0.029**	**0.339**	**81**	**0.021**	*
Adults Only	0.561	-0.127	0.216	13	0.584	

Table S2 | Task Differences in Visual Word Form Area Size
Results from a two-tailed, paired-samples t-test comparing the size of the VWFA when drawn using only one-back task data and only fixation task data. Significant results are displayed in bold and asterisks indicate the degree of significance ($p < 0.001$: ***, $p < 0.01$: **, $p < 0.05$: *)

BOLD Response ~ Category * ROI Task * Map Task + (1 | Participant)	β	Std. Err	DOF	t	p	
Intercept (ROI Task: One-back | Map Task: One-back | Text)	**1.136**	**0.067**	**175.457**	**16.912**	**1.09E-38**	***
Category: Pseudofonts	**-0.397**	**0.057**	**1400.672**	**-7.030**	**3.21E-12**	***
Category: Faces	**-0.943**	**0.057**	**1400.672**	**-16.691**	**3.49E-57**	***
Category: Objects	**-0.712**	**0.057**	**1400.672**	**-12.598**	**1.49E-34**	***
Category: Limbs	**-0.629**	**0.057**	**1400.672**	**-11.127**	**1.28E-27**	***
ROI Task: Fixation	0.094	0.058	1401.675	1.623	0.105	
Map Task: Fixation	**-0.254**	**0.057**	**1400.672**	**-4.500**	**7.35E-06**	***
Category: Pseudofonts * ROI Task: Fixation	0.146	0.082	1400.672	1.784	0.075	.
Category: Faces * ROI Task: Fixation	0.047	0.082	1400.672	0.582	0.561	
Category: Objects * ROI Task: Fixation	**0.164**	**0.082**	**1400.672**	**2.016**	**0.044**	*
Category: Limbs * ROI Task: Fixation	0.139	0.082	1400.672	1.708	0.088	.
Category: Pseudofonts * Map Task: Fixation	0.030	0.080	1400.672	0.369	0.712	
Category: Faces * ROI Map: Fixation	**0.238**	**0.080**	**1400.672**	**2.978**	**0.003**	**
Category: Objects * ROI Map: Fixation	**0.249**	**0.080**	**1400.672**	**3.117**	**0.002**	**
Category: Limbs * ROI Map: Fixation	**0.161**	**0.080**	**1400.672**	**2.010**	**0.045**	*
ROI Task: Fixation * Map Task: Fixation	**0.174**	**0.082**	**1400.672**	**2.128**	**0.033**	*
Category: Pseudofonts * ROI Task: Fixation * Map Task: Fixation	**-0.323**	**0.115**	**1400.672**	**-2.800**	**0.005**	**
Category: Faces * ROI Task: Fixation * Map Task: Fixation	**-0.312**	**0.115**	**1400.672**	**-2.706**	**0.007**	**
Category: Objects * ROI Task: Fixation * Map Task: Fixation	**-0.307**	**0.115**	**1400.672**	**-2.658**	**0.008**	**
Category: Limbs * ROI Task: Fixation * Map Task: Fixation	**-0.303**	**0.115**	**1400.672**	**-2.623**	**0.009**	**

a

BOLD Response ~ ROI Task * Map Task + (1 | Participant)	β	Std. Err	DOF	t	p	
**Text**						
Intercept (ROI Task: One-back | Map Task: One-back)	**1.132**	**0.070**	**124.219**	**16.184**	**2.21E-32**	***
ROI Task: Fixation	0.088	0.055	218.884	1.585	0.114	
Map Task: Fixation	**-0.254**	**0.053**	**216.516**	**-4.754**	**3.63E-06**	***
ROI Task: Fixation * Map Task: Fixation	**0.174**	**0.077**	**216.516**	**2.249**	**0.026**	*
**Pseudofonts**						
Intercept (ROI Task: One-back | Map Task: One-back)	**0.738**	**0.070**	**132.021**	**10.532**	**3.33E-19**	***
ROI Task: Fixation	**0.245**	**0.059**	**218.197**	**4.124**	**5.30E-05**	***
Map Task: Fixation	**-0.225**	**0.057**	**215.426**	**-3.919**	**1.19E-04**	***
ROI Task: Fixation * Map Task: Fixation	-0.149	0.083	215.426	-1.805	0.072	.
**Faces**						
Intercept (ROI Task: One-back | Map Task: One-back)	**0.189**	**0.057**	**134.087**	**3.337**	**0.001**	**
ROI Task: Fixation	**0.150**	**0.048**	**219.062**	**3.110**	**0.002**	**
Map Task: Fixation	-0.016	0.047	216.269	-0.350	0.727	
ROI Task: Fixation * Map Task: Fixation	**-0.138**	**0.067**	**216.269**	**-2.055**	**0.041**	*
**Objects**						
Intercept (ROI Task: One-back | Map Task: One-back)	**0.428**	**0.070**	**130.137**	**6.134**	**9.57E-09**	***
ROI Task: Fixation	**0.248**	**0.058**	**218.953**	**4.276**	**2.84E-05**	***
Map Task: Fixation	-0.005	0.056	216.324	-0.093	0.926	
ROI Task: Fixation * Map Task: Fixation	-0.133	0.081	216.324	-1.647	0.101	
**Limbs**						
Intercept (ROI Task: One-back | Map Task: One-back)	**0.508**	**0.068**	**124.951**	**7.495**	**1.06E-11**	***
ROI Task: Fixation	**0.236**	**0.054**	**218.190**	**4.333**	**2.25E-05**	***
Map Task: Fixation	-0.094	0.053	215.734	-1.780	0.076	.
ROI Task: Fixation * Map Task: Fixation	-0.129	0.076	215.734	-1.699	0.091	.

b

Table S3 | LME Results for Activation by Split Task VWFA on Split Task Data
a model results for a three way interaction between category, ROI task, and map task **b** model results for a two way interaction between ROI task and map task ran separately for each category. Significant results are displayed in bold and asterisks indicate the degree of significance ($p < 0.001$: ***, $p < 0.01$: **, $p < 0.05$: *)

Text Selectivity ~ ROI Task * Map Task + (1 | Participant)						
	**β**	**Std. Err**	**DOF**	**t**	**p**	
Intercept (ROI Task: One-back | Map Task: One-back)	**0.212**	**0.013**	**287.023**	**16.100**	**5.32E-42**	***
ROI Task: Fixation	**-0.081**	**0.018**	**227.418**	**-4.510**	**1.04E-05**	***
Map Task: Fixation	**-0.058**	**0.018**	**220.218**	**-3.274**	**0.001**	**
ROI Task: Fixation * Map Task: Fixation	**0.137**	**0.025**	**220.218**	**5.400**	**1.72E-07**	***

Table S4 | LME Results for Text Selectivity by Split Task VWFA on Split Task Data model results for a two way interaction between ROI task and map task. Significant results are displayed in bold and asterisks indicate the degree of significance ($p < 0.001$: ***, $p < 0.01$: **, $p < 0.05$: *)

BOLD Response ~ ROI + (1 | Participant)						
	β	Std. Err	DOF	t	p	
**Text**						
Intercept (native VWFA)	**1.069**	**0.050**	**260.016**	**21.366**	**3.60E-59**	***
cVWFA	**-0.171**	**0.050**	**368.214**	**-3.452**	**6.21E-04**	***
aVWFA	**-0.478**	**0.050**	**368.214**	**-9.648**	**8.75E-20**	***
rVWFA	**-0.197**	**0.050**	**368.214**	**-3.989**	**7.99E-05**	***
kVWFA	-0.039	0.050	368.214	-0.788	0.431	
**Pseudofonts**						
Intercept (native VWFA)	**0.715**	**0.046**	**279.215**	**15.456**	**2.31E-39**	***
cVWFA	0.085	0.047	368.567	1.808	0.071	.
aVWFA	**-0.180**	**0.047**	**368.567**	**-3.818**	**1.58E-04**	***
rVWFA	**0.157**	**0.047**	**368.567**	**3.317**	**0.001**	**
kVWFA	**0.261**	**0.047**	**368.567**	**5.525**	**6.23E-08**	***
**Faces**						
Intercept (native VWFA)	**0.270**	**0.054**	**203.838**	**5.022**	**1.11E-06**	***
cVWFA	**0.502**	**0.047**	**366.619**	**10.716**	**1.72E-23**	***
aVWFA	**0.241**	**0.047**	**366.619**	**5.153**	**4.20E-07**	***
rVWFA	**0.561**	**0.047**	**366.619**	**11.981**	**3.87E-28**	***
kVWFA	**1.066**	**0.047**	**366.619**	**22.754**	**4.32E-72**	***
**Objects**						
Intercept (native VWFA)	**0.578**	**0.060**	**204.162**	**9.574**	**3.59E-18**	***
cVWFA	**0.530**	**0.053**	**366.400**	**10.044**	**3.98E-21**	***
aVWFA	**0.213**	**0.053**	**366.400**	**4.027**	**6.86E-05**	***
rVWFA	**0.747**	**0.053**	**366.400**	**14.147**	**1.48E-36**	***
kVWFA	**0.946**	**0.053**	**366.400**	**17.933**	**4.50E-52**	***
**Limbs**						
Intercept (native VWFA)	**0.613**	**0.061**	**201.459**	**10.003**	**2.22E-19**	***
cVWFA	**0.589**	**0.053**	**366.113**	**11.068**	**9.34E-25**	***
aVWFA	**0.278**	**0.053**	**366.113**	**5.220**	**3.00E-07**	***
rVWFA	**0.765**	**0.053**	**366.113**	**14.382**	**1.71E-37**	***
kVWFA	**0.989**	**0.053**	**366.113**	**18.582**	**9.19E-55**	***

a

b

BOLD Response ~ ROI + (1 | Participant)	β	Std. Err	DOF	t	p	
**Text**						
Intercept (native FFA)	**0.649**	**0.035**	**136.897**	**18.743**	**1.28E-39**	***
cFFA	**0.145**	**0.023**	**380.000**	**6.395**	**4.72E-10**	***
aFFA	**0.259**	**0.023**	**380.000**	**11.412**	**3.87E-26**	***
rFFA	-0.011	0.023	380.000	-0.493	0.622	
kFFA	0.015	0.023	380.000	0.682	0.496	
**Pseudofonts**						
Intercept (native FFA)	**0.567**	**0.031**	**144.616**	**18.242**	**2.49E-39**	***
cFFA	**0.169**	**0.022**	**380.000**	**7.766**	**7.61E-14**	***
aFFA	**0.290**	**0.022**	**380.000**	**13.341**	**1.45E-33**	***
rFFA	-0.011	0.022	380.000	-0.498	0.618	
kFFA	0.018	0.022	380.000	0.840	0.402	
**Faces**						
Intercept (native FFA)	**1.714**	**0.043**	**150.468**	**40.009**	**4.37E-82**	***
cFFA	**-0.326**	**0.031**	**380.000**	**-10.455**	**1.18E-22**	***
aFFA	**-0.223**	**0.031**	**380.000**	**-7.125**	**5.25E-12**	***
rFFA	**-0.657**	**0.031**	**380.000**	**-21.053**	**9.06E-66**	***
kFFA	**-0.455**	**0.031**	**380.000**	**-14.565**	**1.71E-38**	***
**Objects**						
Intercept (native FFA)	**1.319**	**0.047**	**136.717**	**27.929**	**2.36E-58**	***
cFFA	**0.175**	**0.031**	**380.000**	**5.653**	**3.10E-08**	***
aFFA	**0.327**	**0.031**	**380.000**	**10.603**	**3.49E-23**	***
rFFA	**-0.226**	**0.031**	**380.000**	**-7.311**	**1.58E-12**	***
kFFA	-0.014	0.031	380.000	-0.449	0.654	
**Limbs**						
Intercept (native FFA)	**1.397**	**0.046**	**131.613**	**30.425**	**2.10E-61**	***
cFFA	**0.070**	**0.028**	**380.000**	**2.464**	**0.014**	*
aFFA	**0.231**	**0.028**	**380.000**	**8.114**	**6.85E-15**	***
rFFA	**-0.240**	**0.028**	**380.000**	**-8.411**	**8.29E-16**	***
kFFA	**-0.086**	**0.028**	**380.000**	**-3.011**	**0.003**	**

Table S5 | Group and Template ROI Activation LME Results
a model results for a estimating stimus percent signal change as a function of VWFA **b** model results for a estimating stimus percent signal change as a function of FFA. Significant results are displayed in bold and asterisks indicate the degree of significance (p < 0.001: ***, p < 0.01: **, p < 0.05: *)

Text Selectivity ~ ROI + (1 | Participant)						
	β	Std. Err	DOF	t	p	
Intercept (native VWFA)	**0.162**	**0.008**	**256.929**	**20.868**	**3.06E-57**	***
cVWFA	**-0.185**	**0.008**	**367.053**	**-24.140**	**8.82E-78**	***
aVWFA	**-0.189**	**0.008**	**367.053**	**-24.704**	**4.60E-80**	***
rVWFA	**-0.221**	**0.008**	**367.053**	**-28.803**	**3.20E-96**	***
kVWFA	**-0.238**	**0.008**	**367.053**	**-31.000**	**1.56E-104**	***

Table S6 | Group and Template ROI Text Selectivity LME Results
Model results for a estimating text selectivity as a function of VWFA. Significant results are displayed in bold and asterisks indicate the degree of significance ($p < 0.001$: ***, $p < 0.01$: **, $p < 0.05$: *)
